# Improving reading skills in students with dyslexia: the efficacy of a sublexical training with rhythmic background

**DOI:** 10.3389/fpsyg.2015.01510

**Published:** 2015-10-06

**Authors:** Silvia Bonacina, Alice Cancer, Pier Luca Lanzi, Maria Luisa Lorusso, Alessandro Antonietti

**Affiliations:** ^1^Department of Psychology, Catholic University of the Sacred HeartMilan, Italy; ^2^Department of Electronics, Information and Bioengineering, Polytechnic UniversityMilan, Italy; ^3^IRCCS “Eugenio Medea”Bosisio Parini, Italy

**Keywords:** reading, developmental dyslexia, music, rhythm, auditory processing, intervention

## Abstract

The core deficit underlying developmental dyslexia (DD) has been identified in difficulties in dynamic and rapidly changing auditory information processing, which contribute to the development of impaired phonological representations for words. It has been argued that enhancing basic musical rhythm perception skills in children with DD may have a positive effect on reading abilities because music and language share common mechanisms and thus transfer effects from the former to the latter are expected to occur. A computer-assisted training, called Rhythmic Reading Training (RRT), was designed in which reading exercises are combined with rhythm background. Fourteen junior high school students with DD took part to 9 biweekly individual sessions of 30 min in which RRT was implemented. Reading improvements after the intervention period were compared with ones of a matched control group of 14 students with DD who received no intervention. Results indicated that RRT had a positive effect on both reading speed and accuracy and significant effects were found on short pseudo-words reading speed, long pseudo-words reading speed, high frequency long words reading accuracy, and text reading accuracy. No difference in rhythm perception between the intervention and control group were found. Findings suggest that rhythm facilitates the development of reading skill because of the temporal structure it imposes to word decoding.

## Introduction

Developmental dyslexia (DD) is a specific learning disorder which has a neurobiological origin and is characterized by the presence of reading difficulties not accounted for by sensory, neurological, or intellectual deficits (Lyon et al., 2003; Snowling and Hulme, 2012). The primary deficit underlying DD is still under debate. Most likely, a combination of different factors is involved (Ramus, 2003; Pennington, 2006; Menghini et al., 2010). However, a specific impairment in phonological processing is still widely assumed to be the core deficit underlying DD, as confirmed by many substantive studies (Goswami, 2000; Snowling, 2000; Lyon et al., 2003; Ramus et al., 2003; Démonet et al., 2004; Ramus and Szenkovits, 2008; Fraser et al., 2010). Phonological representation dysfunction is associated with difficulties in dynamic and rapidly changing auditory information processing (Tallal and Stark, 1981; Tallal et al., 1985, 1993; Farmer and Klein, 1995; Breier et al., 2003; Tallal, 2004; Corriveau et al., 2007; Huss et al., 2011). Children with DD also lack sensitivity to speech prosody (Corriveau et al., 2007; Goswami et al., 2010), pitch perception (Baldeweg et al., 1999; Goswami et al., 2011) and rhythm (Overy et al., 2003; Thomson and Goswami, 2008; Corriveau and Goswami, 2009; Huss et al., 2011; Goswami et al., 2013; Flaugnacco et al., 2014), which are sound attributes cued by amplitude, duration, and frequency changes of the acoustic signal. These basic auditory perceptual and timing difficulties could contribute to the development of impaired phonological representation for words, which is the distinctive feature of DD (Leong and Goswami, 2014a,b). The struggle experienced by people with DD with rapid temporal processing of acoustic information concerns both speech (Tallal and Stark, 1981) and non-speech sounds (Breier et al., 2001; Cantiani et al., 2009), such as music (Huss et al., 2011). Neuroimaging studies provide evidence of a significant overlap in the brain regions involved in processing the characteristics of both auditory speech and non-speech signals (Joanisse and Gati, 2003; Brown et al., 2006; Musacchia et al., 2007; Schön et al., 2010; Abrams et al., 2011; James, 2012; Jantzen et al., 2014). Many studies also report an association between music and reading skills. Musical expertise has a positive effect on language and literacy abilities in normal-reading children (Hurwitz et al., 1975; Douglas and Willats, 1994; Anvari et al., 2002; Forgeard et al., 2008; Moreno et al., 2009; Degé and Schwarzer, 2011; Brandt et al., 2012). Furthermore, in both normal-reading children and children with DD, music discrimination abilities, assessed using tonal-melodic and rhythmic tasks, predict phonological and reading skills (Forgeard et al., 2008). All these findings together suggest that interventions aimed at enhancing basic auditory perception skills of children with DD may impact language and reading abilities.

Some conjectures about how music could help children with DD have been put forward (Overy, 2003; Tallal and Gaab, 2006). Changes caused by music expertise might transfer to other domains involving the processing of pitch, timing, and timbre cues, such as verbal language. So far, only two studies investigated the effectiveness of music training as a remediation for children with DD using pre vs. post-training designs; the majority of the studies aimed at testing the effects of musical interventions considered poor readers or preschoolers, who did not receive a proper diagnosis of DD. Overy (2003) examined the influence of a 15-week music training on reading skills of 9 children with DD from two different schools (mean age 8.8 years old). Music lessons were conducted three times per week in sessions of 20 min each. Reading skills, as well as phonological, spelling, and music abilities, were assessed across the music intervention period and the preceding 15-week control period using the WORD tests of single word reading and spelling (Wechsler, 1993), selected tests from the Phonological Abilities Test (PAT: Muter et al., 1997), the Dyslexia Early Screening Test (DEST: Nicolson and Fawcett, 1996) and an original collection of musical aptitude tests developed *ad hoc*. A significant improvement in phonological, spelling, rhythm copying, and rapid auditory processing skills emerged; however, reading skills were not significantly affected by the musical treatment. Register et al. (2007) measured the efficacy of a short-term music curriculum on reading skills of 33 second-grade students and 8 students who had been identified as having a specific learning disability (SLD) in reading. The curriculum was implemented in 18 sessions of 30 min each. It was designed to target reading comprehension and vocabulary skills. Reading skills were evaluated pre and post curriculum intervention via the Vocabulary and Reading Comprehension subtests of the GMRT (MacGinitie et al., 2000) for second grade. Significant improvements in word decoding, word knowledge, and reading comprehension were found after the music intervention in the group of children with SLD.

Although some promising outcomes have been reported, a meta-analysis by Cogo-Moreira et al. (2012) pointed out the lack of well-designed studies. The authors screened 876 studies regarding the effectiveness of music education on reading skills in children and adolescents with dyslexia. Only six studies among these had the potential to be included in the meta-analysis since they mentioned musical education for children with DD (Fiveash, 1995; Jaarsma et al., 1998; Banks, 1999; Overy, 2003; Draper, 2007; Register et al., 2007). However, all were ultimately excluded either because of the lack of randomized controlled trials (including quasi-randomized or cluster-randomized trials) or because they did not use any reading skills' measure as an outcome. According to the authors of the meta-analysis, further research via randomized controlled trials is needed. Even though the hypothesis of a positive effect of music training on the impaired auditory perception and timing processing of children with DD is supported by much evidence, it appears that music education cannot solely produce improvements in reading skills comparable with those resulting from traditional intervention methods for DD, which therefore should not be replaced (Kraus and Chandrasekaran, 2010). Thus, combining traditional intervention aimed at enhancing grapheme-phoneme connections and music intervention appears to be an interesting and innovative approach. A meta-analysis carried out by considering interventions implemented in Italy (Tressoldi et al., 2003) suggested that the most effective treatments for DD are interventions aimed at improving the automatic decoding of sublexical and lexical stimuli, such as the Sublexical Treatment (Cazzaniga et al., 2005) and the visual hemispheric-specific stimulation inspired by the Balance-Model (Bakker et al., 1995; Lorusso et al., 2006, 2011).

Taking these findings into account, a research program was designed to devise and test a treatment which combines a traditional remediation approach (sublexical treatment) with rhythm processing training. A rhythmic accompaniment provides readers a structure which helps them to organize temporal cues of speech sounds (Chandrasekaran et al., 2009). Rhythm should therefore assume the role of an aid in rapid auditory processing which can support decoding ability.

The purpose of the present study is to evaluate the effectiveness of the computer-assisted version of a new intervention, called Rhythmic Reading Training (RRT), aimed at improving reading skills in students with DD, in which reading exercises are combined with rhythm processing. We expected that participation in the designed intervention would increase reading speed and accuracy in a group of Italian students with DD, as well as enhancing rhythm perception abilities.

## Materials and methods

### Design

In order to evaluate the efficacy of RRT as a treatment for dyslexia, a test-training-retest experimental design was applied. We measured possible changes in reading skills of a group of children with DD between the beginning (pre phase) and the end of the intervention period (post phase). Reading improvements were compared to those of a control group of children with DD matched for gender, school grade, and level of reading impairment. Reading skills of the control group were monitored before and after a period of the same length of the intervention during which no specific activity addressed to improve reading skills was carried out.

### Participants

Twenty-eight students aged between 11 and 14 years (mean age = 12.07 years, *SD* = 1.14) with DD participated in the study. They attended a junior high school in Lecco (Lombardy, Northern Italy) and had been previously diagnosed with DD on the basis of standard inclusion and exclusion criteria (ICD-10: World Health Organization, 1992) and of the ordinary diagnosis procedure followed in the Italian context. Also, their diagnoses of DD were updated at least 6 months before the participation in the study either by the National Health System or private accredited centers. Students who had comorbidities with other psychiatric or psychological disorders were excluded from the study. All participants shared similar cultural and socio-economic status as they all belonged to similar living environments.

The parents of all students diagnosed with DD were contacted by the researchers. The purpose and procedures of the study were explained. Parental consent was obtained for all students, who agreed to take part to the study.

The entire research process was conducted according to standards of the Helsinki Declaration. Two subgroups of the same size, matched for gender, school grade, and level of reading impairment were created and randomly assigned either to the intervention or the control condition (Table 1).

**Table 1:** **Participants' characteristics**.

**Group**	**Control group (*N* = 14)** **Mean (*SD*)**	**Intervention group (*N* = 14)** **Mean (*SD*)**	**Group comparison** ***t*_(26)_, *p***
Male	10	10	
Female	4	4	
First grade	2	2	
Second grade	6	6	
Third grade	6	6	
READING Speed^a^	1.38 (0.68)	1.06 (1.23)	−0.85, 0.400
READING Accuracy^a^	3.02 (2.51)	2.11 (2.38)	0.98, 0.320
Rhythm reproduction^b^	3.93 (2.52)	4.14 (3.78)	−0.17, 0.861

a *Scores are expressed as z-scores in the text reading task (positive z-scores represent a performance below average, negative z-scores a performance above average)*.

b *Scores are expressed as number of errors performed in the rhythm reproduction tasks*.

### Assessment

Reading and rhythmic perception skills were assessed before and after the intervention or control period. The assessment of reading skills was carried out throughout two different batteries of tests. —“Prova di lettura di parole e non parole” (Word and pseudo-word reading test: Zoccolotti et al., 2005), in which speed and accuracy scores were computed for single word (four lists of 30 words each with different lengths and frequency of use) and pseudo-word reading (two lists of 30 pseudo-words each with different lengths).—“Nuove prove di lettura MT per la scuola media inferiore” (New MT reading tests for junior high school: Cornoldi and Colpo, 1995), a set of tests providing accuracy and speed scores in reading aloud age-normed texts. Length and complexity of texts were standardized for each semester of school year. Both batteries are the most commonly used in Italy to assess reading abilities in students with DD. For both batteries, z-scores for reading accuracy and reading speed were computed from raw scores (respectively, the number of errors and total reading time expressed in seconds). A decrease in speed and accuracy measures corresponds to an improvement of the reading performance. Because the training specifically targeted the decoding aspects of reading, no measures of reading comprehension were included in the testing. Rhythm perception ability—which might be improved thanks to the rhythmic component of RRT—was assessed, both before and after the treatment or control period, through the rhythm reproduction task (Stambak, 1951). This consists in the request to reproduce a set of rhythmic patterns of increasing complexity performed by the examiner. Scores are computed by counting the number of errors in the reproduction of rhythmic patterns. The pre- and post-intervention assessment of rhythm skills was aimed at allowing us to understand if the effects of RRT are specific to the language or involve a general improvement in rhythm discrimination and reproduction abilities.

### Training

RRT is a child-friendly computerized reading program addressed to Italian students with DD aged 8–13 (Cancer and Antonietti, 2011; Germagnoli et al., in press). The training program is composed of three categories of exercises designed to improve reading skills. Each category of exercises is aimed at training a specific reading ability. The section “Syllables” trains syllable recognition. The section “Merging” involves merging syllables for creating words. The goal of the section “Words and Pseudo-words” is to train word, pseudo-word, and small phrases decoding.

All reading exercises include a rhythmical accompaniment with gradually increasing speed. Students are taught to read the verbal stimuli (i.e., syllables, words, pseudo-words, phrases) presented on the screen in synchrony with the rhythmic accompaniment. The first time an exercise is presented, the stimulus (or the part of the stimulus) which had to be read is indicated by a visual cue (consisting of highlighting the target grapheme in red) synchronized with musical rhythm, so to allow students to understand clearly in which manner they had to read the verbal materials.

All participants were trained using the full range of exercises. The speed of presentation of the verbal stimuli in the exercises was dependent upon the reading level of each participant. The rate of increase of the speed of the exercises, by moving from the starting presentation to the subsequent ones, was proportionally the same for all participants in the training.

### Procedure

All tests included in the assessment were administered individually in a quiet room by an examiner who was an expert in reading assessment. Tests were presented in a single session. The examiner was blind to the condition to which the examined student belonged. Participants assigned to the intervention condition took part in the training program for 9 biweekly sessions each of 30 min in length, resulting in a total of 4.5 h of intervention. Training sessions were individual and were managed by the same researcher in a quite room of the school. During the training session the child sat in front of the computer and performed the proposed reading exercises under the supervision of the researcher. The number of exercises performed in each session varied according to the difficulty and the speed of the exercises. All the tasks were repeated at least three times at gradually increasing speed. The researcher set the speed at which stimuli had been presented according to the student's performance in each exercise. The student had to fulfill at least a reading accuracy of 95% of the verbal stimuli in each exercise in order to speed up and/or proceed to the next exercise.

## Results

A mixed factorial ANOVA was carried out in order to evaluate the effect of RRT on reading accuracy and reading speed. Condition (intervention vs. control) was considered as the independent between-subject variable; phase (pre vs. post) as the independent within-subject variable.

**Figure 1:**
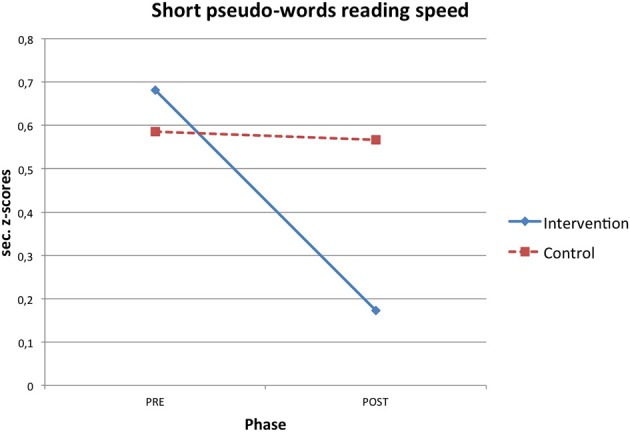
**Phase X condition interaction effect for short pseudo-word reading speed z-scores**.

**Figure 2:**
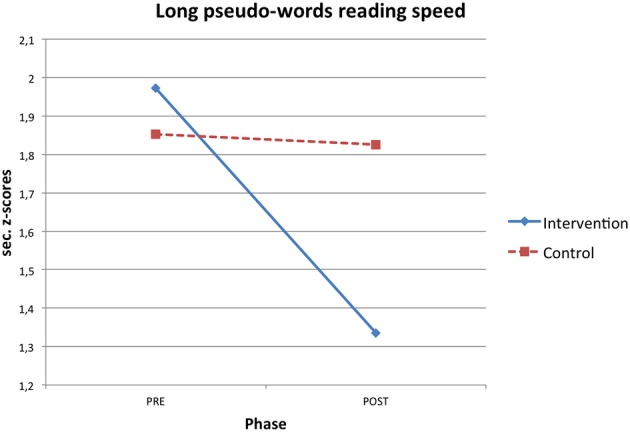
**Phase X condition interaction effect for long pseudo-word reading speed z-scores**.

**Figure 3:**
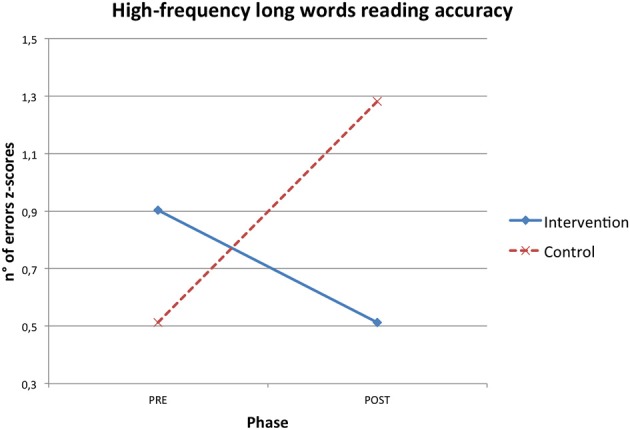
**Phase X condition interaction effect for high frequency long word reading accuracy z-scores**.

**Figure 4:**
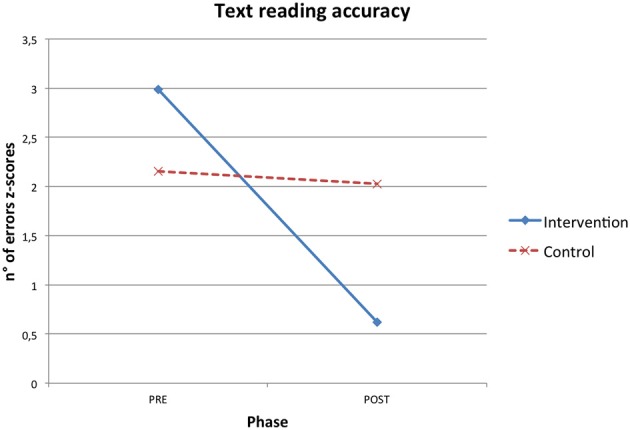
**Phase X condition interaction effect for text reading accuracy z-scores**.

RTT improved participants' reading skills. Both reading speed and accuracy mean z-scores increased after the intervention and these gains were significantly higher in the intervention than in the control condition (see Table 2). Significant interaction effects were found in short pseudo-words reading speed (η^2^ = 0.145), long pseudo-words reading speed (η^2^ = 0.224), high frequency long words reading accuracy (η^2^ = 0.172) and text reading accuracy (η^2^ = 0.278; Figures 1–4).

**Table 2:** **Mean z-scores (SD in parentheses) and ANOVA results for words, pseudo-words and text reading speed, and accuracy tasks (*p*-values indicating significant effects are marked in bold)**.

		**Control group** **Mean(*SD*)**	**Intervention group** **Mean (*SD*)**	**Phase main effect *F*_(1, 26)_, *p***	**Phase X condition interaction effect *F*_(1, 26)_, *p***
Short pseudo-words speed	PRE	0.58 (1.24)	0.68 (1.20)	5.140, **0.032**	4.411, **0.046**
	POST	0.57 (1.17)	0.17 (0.95)		
Short pseudo-words accuracy	PRE	0.68 (1.33)	0.79 (1.58)	2.364, 0.136	2.364, 0.136
	POST	0.38 (1.31)	0.27 (0.81)		
Long pseudo-words speed	PRE	1.85 (1.65)	1.97 (1.69)	8.836, **0.006**	7.493, **0.011**
	POST	1.82 (1.75)	1.22 (1.34)		
Long pseudo-words accuracy	PRE	0.56 (1.07)	1.08 (1.48)	1.636, 0.212	2.059, 0.163
	POST	0.60 (1.30)	0.45 (1.01)		
High frequency short words speed	PRE	−0.16 (0.74)	0.04 (1.07)	2.634, 0.116	0.474, 0.497
	POST	−0.27 (1.08)	−0.20 (0.76)		
High frequency short words accuracy	PRE	0.64 (1.83)	0.80 (1.86)	2.737, 0.110	1.020, 0.322
	POST	0.38 (1.71)	−0.25 (0.60)		
High frequency long words speed	PRE	1.31 (1.95)	1.44 (1.69)	2.38, 0.135	0.313, 0.581
	POST	1.11 (1.81)	1,00 (1.66)		
High frequency long words accuracy	PRE	0.51 (1.20)	0.90 (1.14)	0.571, 0.457	5.387, **0.028**
	POST	1.281 (1.21)	0.51 (1.04)		
Low frequency short words speed	PRE	1.11 (1.40)	1.00 (1.43)	0.263, 0.613	0.031, 0.861
	POST	1.00 (1.43)	0.95 (1,30)		
Low frequency short words accuracy	PRE	1.08 (1.63)	0.64 (1.16)	4.793, **0.038**	2.199, 0.150
	POST	0.16 (1.04)	0.46 (1.42)		
Low frequency long words speed	PRE	2.07 (1.70)	1.84 (1.43)	9.174, **0.005**	0.018, 0.895
	POST	1.66 (1.34)	1.46 (1.46)		
Low frequency long words accuracy	PRE	0.38 (1.26)	0.45 (1.40)	8.563, **0.007**	0.352, 0.558
	POST	−0.30 (0.76)	−0.30 (0.76)		
Text reading speed	PRE	1.26 (0.94)	1.18 (1.06)	4.757, **0.038**	0.381, 0.542
	POST	1.09 (0.91)	1.09 (0.92)		
Text reading accuracy	PRE	2.15 (2.75)	2.99 (2.12)	12.427, **0.002**	10.020, **0.004**
	POST	2.03 (2.56)	0.62 (1.21)		

In particular, the time required to read short pseudo-words and long pseudo-words decreased after the intervention respectively of 0.51 and 0.75 z-scores on average. Regarding accuracy, the number of reading errors was reduced of 0.39 z-scores for high frequency long words and of 2.37 z-scores for text on average.

Some significant main effects due to the phase were also found in short pseudo-words reading speed (η^2^ = 0.165), low frequency short words reading accuracy (η^2^ = 0.156), long pseudo-words reading speed (η^2^ = 0.254), low frequency long words reading speed (η^2^ = 0.261), low frequency long words reading accuracy (η^2^ = 0.248), text reading speed (η^2^ = 0.155) and text reading accuracy (η^2^ = 0.323; Table 2). These results suggest that task habituation might have had an effect on reading for those measures.

When a significant interaction effect occurred, additional ANOVAs were computed to test differences between pre and post phase separately within both the control and intervention condition. It emerged that improvements from the pre to the post phase were statistically significant only for the intervention group in short pseudo-words reading speed [*F*_(1, 13)_ = 7.375, *p* = 0.018, η^2^ = 0.362], long pseudo-words reading speed [*F*_(1, 13)_ = 11.970, *p* = 0.004, η^2^ = 0.479] and text reading accuracy [*F*_(1, 13)_ = 25.255, *p* < 0.001, η^2^ = 0.660], while pre-post phase differences were never statistically significant for the control group [short pseudo-words reading speed: *F*_(1, 13)_ = 0.020, *p* = 0.890, η^2^ = 0.002; long pseudo-words reading speed: *F*_(1, 13)_ = 0.043, *p* = 0.838, η^2^ = 0.003; text reading accuracy: *F*_(1, 13)_ = 0.058, *p* = 0.813, η^2^ = 0.004].

Concerning the rhythm reproduction task, no significant difference between the control and the intervention condition was found. After both the control and the intervention period, participants performed the test better: a decrease of 0.93 (*SD* = 2.81) mistakes on average after the control period and a decrease of 2.00 (*SD* = 2.04) mistakes on average after the intervention period were found.

## Discussion and conclusions

The aim of the present study was to test the effect of an innovative computer-assisted training intervention, which combines a traditional remediation approach with rhythm processing, on reading by a group of Italian students with DD. Previous research suggested that musical abilities play a role in reading and that musical training might improve reading skills. However, the musical intervention programs which have been tested included a variety of activities (listening, singing, tapping, playing an instrument, and so on) which involved, in isolation or in combination, different processes (pitch discrimination, reproduction of metric patterns etc.). Thus, it is not possible to understand which mechanism is responsible of the observed changes, if any, in reading capacity. The tool which was tested in the present investigation focused instead on only a specific aspect of music: rhythm; and engaged trainees in only a kind of activity, namely, synchronized reading, in which the musical and verbal dimensions are closely intertwined.

First, a positive effect of the treatment on both reading speed and accuracy emerged. Significant improvements after the RRT were found on both short and long pseudo-words reading speed, as well as in high frequency long words and in text reading accuracy. These results suggest that RRT is efficient in boosting accuracy in reading the kind of materials to which students are usually exposed (namely, high frequently used words and text) and in enhancing speed when the grapheme-phoneme conversion mechanism is required, such as in pseudo-word reading.

The effect of the training program seems to be specific on reading skills, as no significant improvement in rhythm reproduction was found. Close relationships between rhythm and reading skills have been reported (Wolff, 2002; Corriveau and Goswami, 2009; Huss et al., 2011; Goswami et al., 2013) supporting the notion that the ability to segregate the continuous flow of perceptual stimuli into distinct units, to identify the alternation of accented and unaccented units, and to process them according to a regular sequence is involved in both music and language. This suggests that the metric structure that rhythm superimposes to verbal materials—as happens in the RRT—facilitates word processing and allows students to assimilate effective reading procedures which can be generalized to new texts.

Considering the duration of the intervention (9 biweekly sessions of 30 min each), far shorter than traditional remediation treatments of DD, the fact that significant improvements of reading skills were found is promising. Results suggest that a combination of reading and rhythmic training could be an effective treatment for dyslexia. Therefore, it seems plausible to expect a stronger improvement after a prolonged RRT intervention.

However, the limited number of participants calls for caution in evaluating the outcomes of the intervention. Another limitation of the study is the use of the same battery of tests for both pre and post phase assessments, especially as the assessment sessions were only 5 weeks apart. This was the best choice to allow a precise comparison of reading abilities. However, this repetition caused a task habituation effects, as showed by significant phase main effects in short pseudo-words reading speed, low frequency short words reading accuracy, long pseudo-words reading speed, low frequency long words reading speed, low frequency long words reading accuracy, text reading speed and text reading accuracy.

In the study no direct assessment of comprehension skills was performed, and this is an additional limitation. It would have been interesting to test if the changes in reading competence induced by RRT concern only word decoding or also comprehension of the corresponding meaning. However, as the training activities were mainly focused on grapheme-to-phoneme conversion skills, with no emphasis on meaning comprehension, we chose to focus our attention only on the decoding components of reading. Assessing possible increases in reading comprehension produced by RRT is worth investigating in the future.

Further research seems to be necessary to validate the effectiveness of RRT. In particular, it would be crucial to study the role of the rhythmic component in reading improvement. Although the hypothesis of a positive effect of music training on the impaired auditory perception and timing processing of children with DD is supported by much evidence, a comparison between RRT and traditional remediation treatments of DD would help to understand the role of music in reading enhancement. Furthermore, a follow-up assessment should be carried out for evaluating the stability of reading improvements.

In conclusion, the combination of music and reading training seems to be a promising strategy for improving reading skills in students with DD. In addition to the effect on reading, this innovative treatment approach also involves an active engagement with music, which provides an enjoyable and pleasant experience for subjects with DD (Antonietti, 2009).

The present findings have both clinical and educational implications. Even though the whole RRT is not implemented fully and in a systematic way, some suggestions can be derived for other kinds of intervention. If traditional intervention programs—involving reading of word subcomponents, spelling and phonemic awareness and so—are carried out, attention paid to the rhythmical aspects both in the input (perception) and output (execution) phases—encompassing extraction of the rhythmic structure of words and syllables and proceeding according to a given temporal pattern—might be beneficial. In school setting teachers could encourage children with DD to first analyze and then read aloud suitable texts (rhymes and poems might be the best candidates) according to a regular rhythmic structure.

### Conflict of interest statement

The authors declare that the research was conducted in the absence of any commercial or financial relationships that could be construed as a potential conflict of interest.
